# Supplementary material for: Population genomics reveals an ancient origin of heartworms in canids
Source: Commun Biol. 2026 Jan 20;9:68. doi: 10.1038/s42003-025-09250-x (PMC12820332; doi:10.1038/s42003-025-09250-x)
Supplement: Supplementary file 1 — Supplementary Information [file 42003_2025_9250_MOESM1_ESM.pdf]

Supplementary Information

Population genomics reveals an ancient origin of heartworms in canids

Supplementary Fig. 1: Genome-wide sequencing coverage per sample (Part 1/2). 2
Supplementary Fig. 2: Genome-wide sequencing coverage per sample (Part 2/2). 3
Supplementary Fig. 3: Sex determination of samples based on relative coverage of sex-linked vs autosomal chromosomes (Part 1/2). 4
Supplementary Fig. 4: Sex determination of samples based on relative coverage of sex-linked vs autosomal chromosomes (Part 2/2). 6
Supplementary Fig. 5: Principal component analysis (PCA) of 218,158 autosomal single-nucleotide polymorphisms (SNPs) from 124 heartworms with labelled metadata. 8
Supplementary Fig. 6: Principal component analysis (PCA) of 124 heartworms from around the world based on the nuclear genome. 9
Supplementary Fig. 7: Genetic relatedness of heartworms based on mitochondrial and Wolbachia endosymbiont variants. 11
Supplementary Fig. 8: Admixture analysis of adult heartworms. 13
Supplementary Fig. 9: Population genetic summary statistics of heartworms collected from diverse hosts. 14
Supplementary Fig. 10: Population genetic summary statistics of heartworms between broad geographical regions. 15
Supplementary Fig. 11: TreeMix analysis of nuclear heartworm data. 17
Supplementary Fig. 12: Summary diagram of the population genomics workflow in this study. 18
Supplementary Fig. 13: Demographic history analysis of heartworms from dogs using a range of generation times. 19

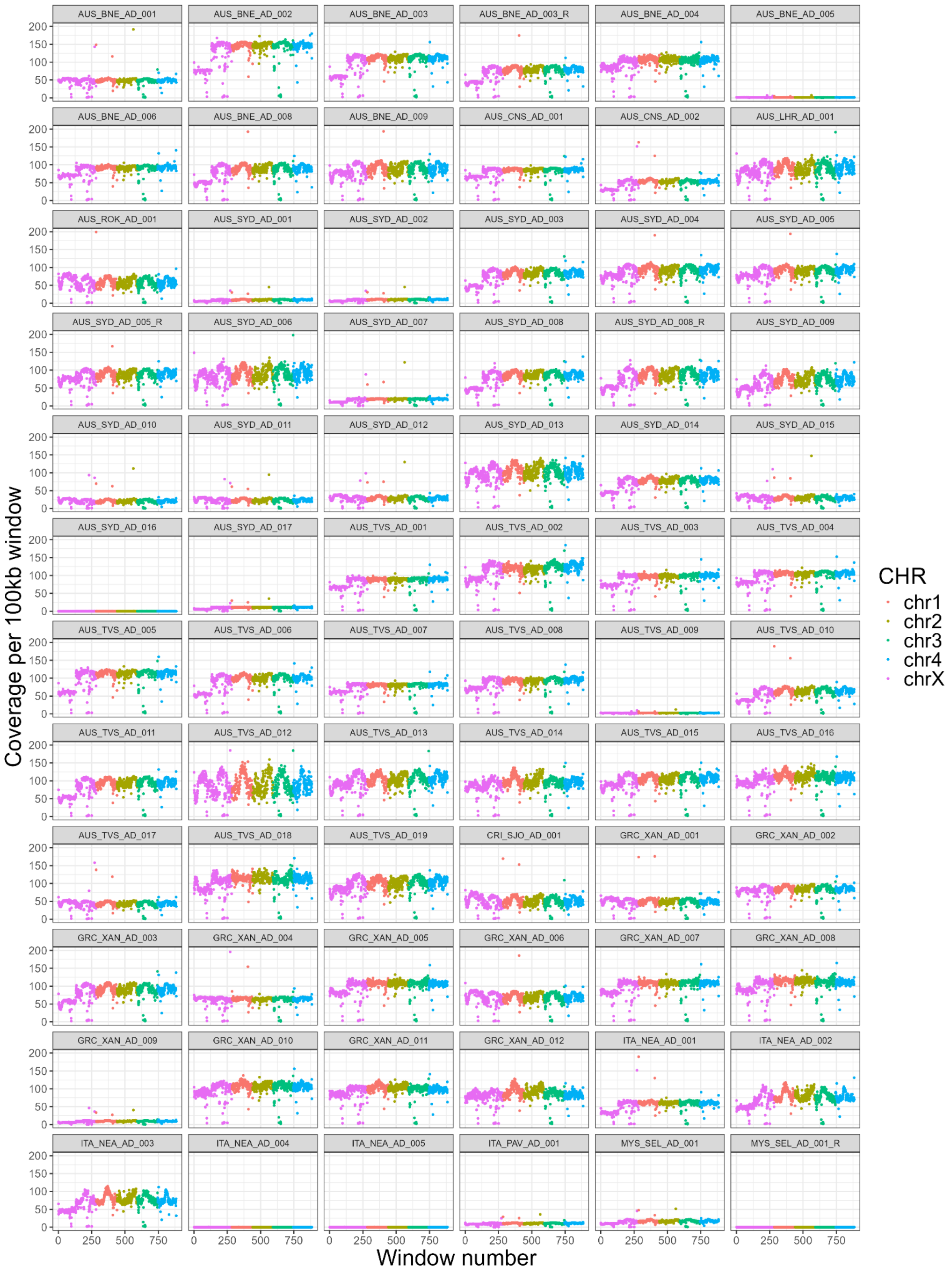

Supplementary Fig. 1: Genome-wide sequencing coverage per sample (Part 1/2).

Coverage was calculated using 100 kb sliding windows for all mapped samples. Each chromosome is shown in a different colour, with a legend on the right. Outliers with coverage >200× were excluded from the plots to enhance visualisation. The remaining samples are shown in Supplementary Fig. 2.

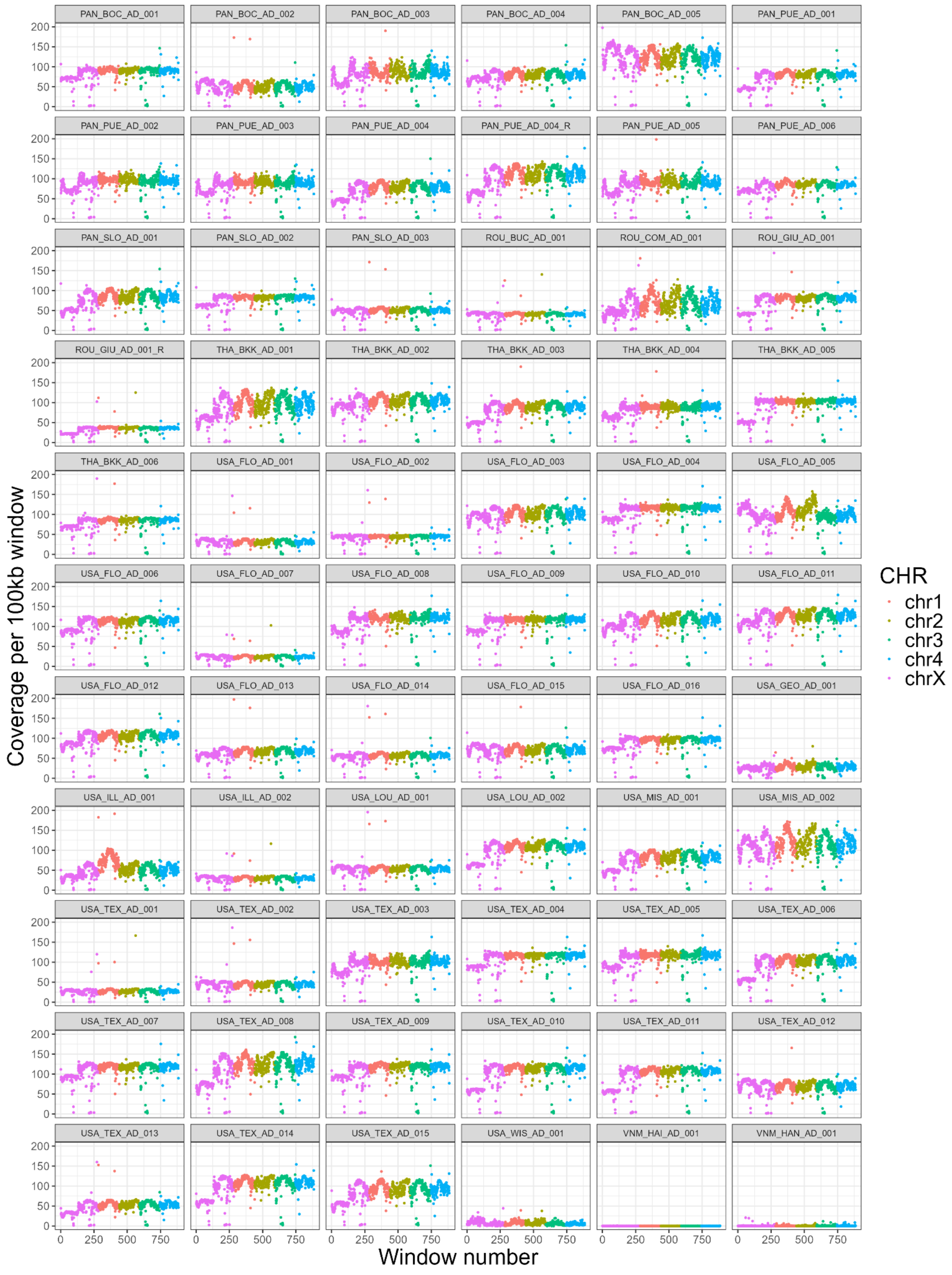

Supplementary Fig. 2: Genome-wide sequencing coverage per sample (Part 2/2).

Coverage was calculated using 100 kb sliding windows for all mapped samples. Each chromosome is shown in a different colour, with a legend on the right. Outliers with coverage >200× were excluded from the plots to enhance visualisation.

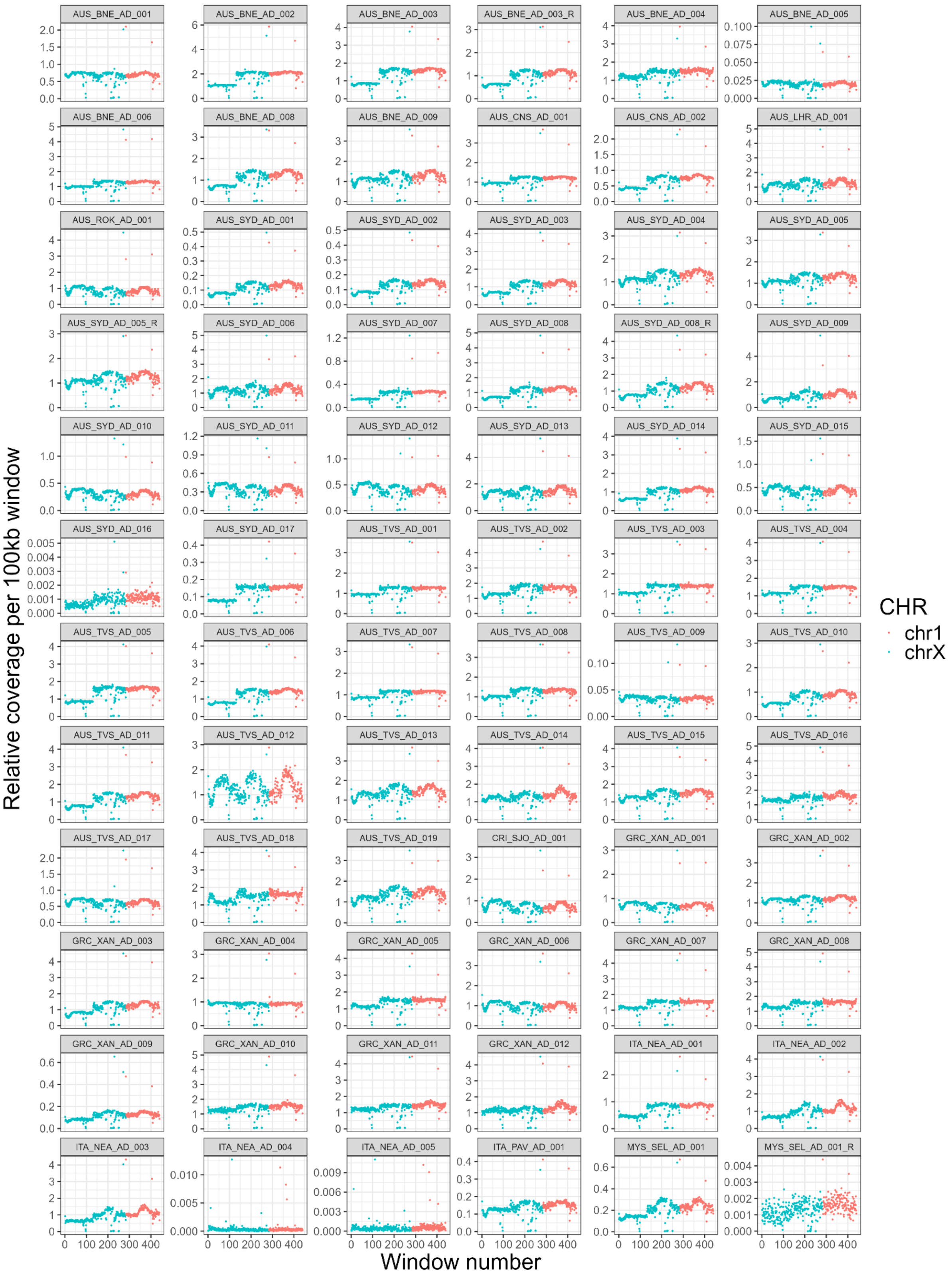

Supplementary Fig. 3: Sex determination of samples based on relative coverage of sex-linked vs autosomal chromosomes (Part 1/2).

Sequencing coverage was assessed using a 100kb sliding window and compared to the overall median coverage. Coverage of the sex-linked X chromosome (blue) was compared to that of an autosome (chromosome 1, red) to determine the parasite's sex. Male canine heartworms show reduced coverage on chromosome X (ratio ~0.5), while females show similar coverage across sex-linked and autosomal chromosomes (ratio ~1). Ambiguous results were observed in samples with low coverage. The remaining samples are shown in Supplementary Fig. 4.

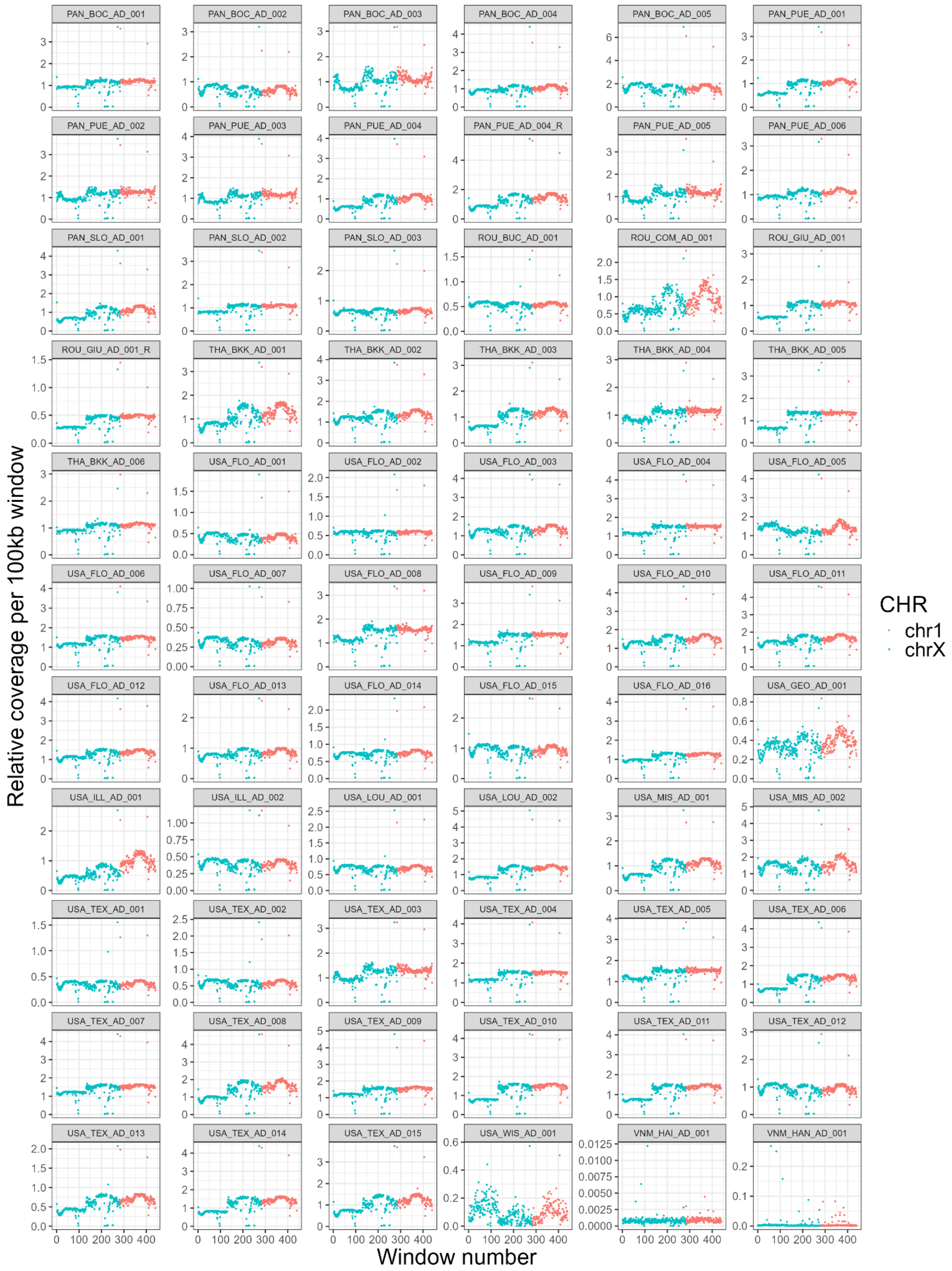

Supplementary Fig. 4: Sex determination of samples based on relative coverage of sex-linked vs autosomal chromosomes (Part 2/2).

Sequencing coverage was assessed using a 100kb sliding window and compared to the overall median coverage. Coverage of the sex-linked X chromosome (blue) was compared to that of an autosome (chromosome 1, red) to determine the parasite’s sex. Male canine heartworms show reduced coverage on chromosome X (ratio ~0.5), while females show similar coverage across sex-linked and autosomal chromosomes (ratio ~1). Ambiguous results were observed in samples with low coverage.

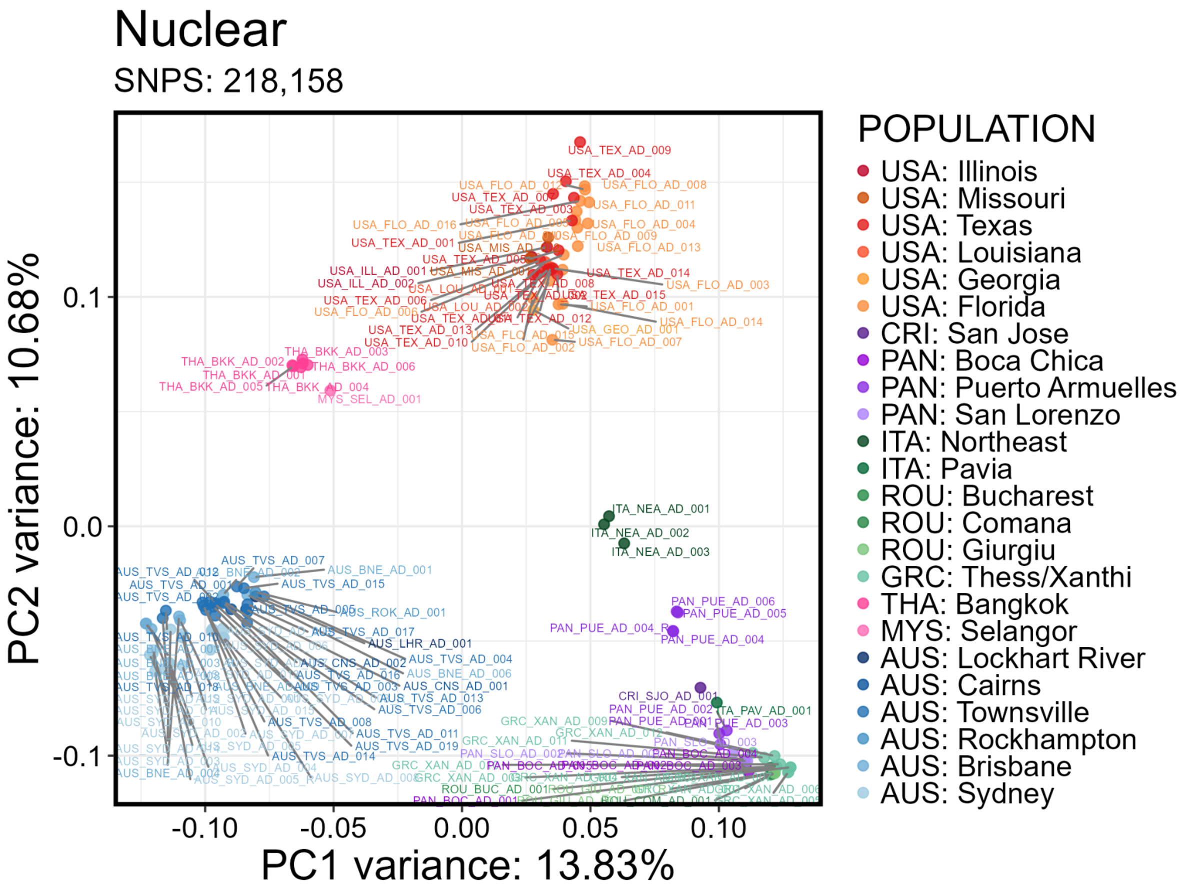

Supplementary Fig. 5: Principal component analysis (PCA) of 218,158 autosomal single-nucleotide polymorphisms (SNPs) from 124 heartworms with labelled metadata.

Samples were collected from the USA, Costa Rica (CRI), Panama (PAN), Italy (ITA), Romania (ROU), Greece (GRC), Thailand (THA), Malaysia (MYS), and Australia (AUS). Replicate samples were included in the analyses. The legend on the right indicates the geographic origin of samples (i.e. COUNTRY: city or region). Principal component 1 (PC1) and principal component 2 (PC2) are shown on the x and y axes, explaining 13.83% and 10.68% of the variation, respectively. These figures are identical to Fig. 1b but include sample labels.

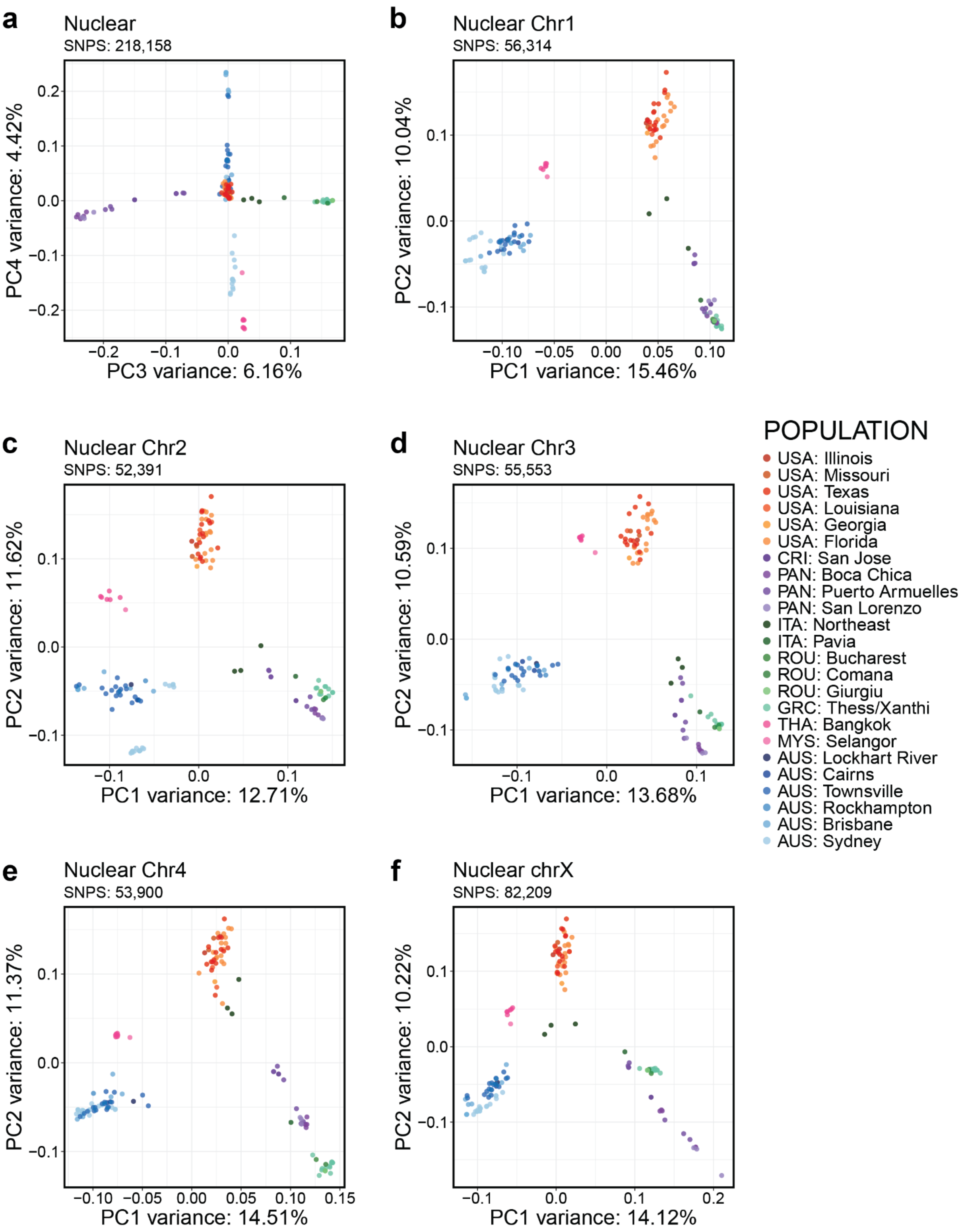

Supplementary Fig. 6: Principal component analysis (PCA) of 124 heartworms from around the world based on the nuclear genome.

Samples were collected from the USA, Costa Rica (CRI), Panama (PAN), Italy (ITA), Romania (ROU), Greece (GRC), Thailand (THA), Malaysia (MYS), and Australia (AUS). Replicate samples were included in the analyses. The legend on the right indicates the geographic origin of samples (i.e. COUNTRY: city or region). The principal components (PCs) are shown on the x and y axes. **a,** PCA of 218,158 autosomal single-nucleotide polymorphisms (SNPs) showing PC3 and PC4, which explained 6.16% and 4.42% of the variation, respectively. **b–f,** PCAs of 52,391 – 82,209 SNPs in chromosome 1, chromosome 2, chromosome 3, chromosome 4, and the sex-linked X-chromosome of canine heartworm. PC1 and PC2 explained 12.71 – 15.46% and 10.04 – 11.62% of the variation, respectively.

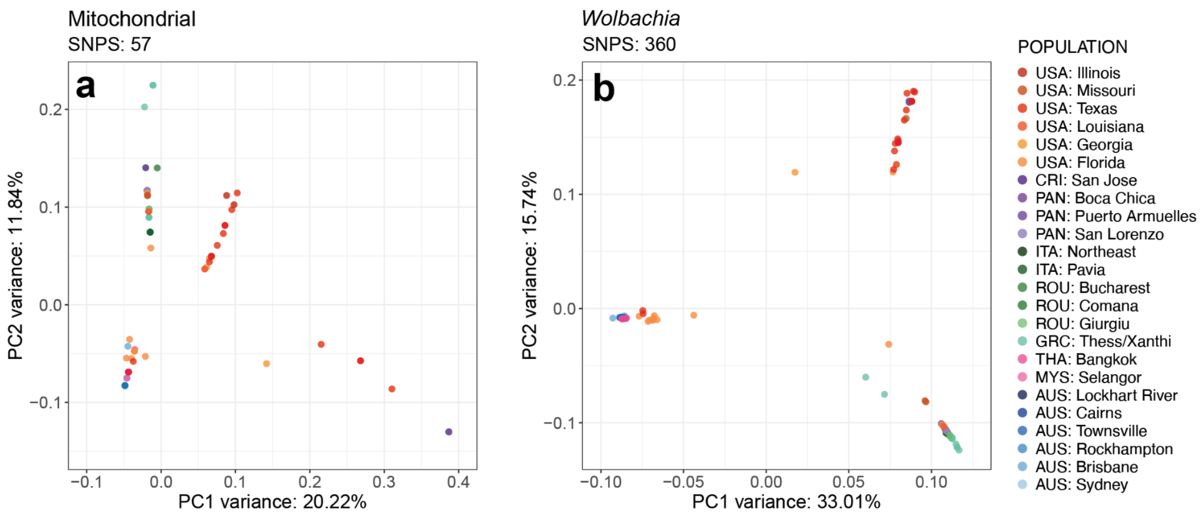

Supplementary Fig. 7: Genetic relatedness of heartworms based on mitochondrial and *Wolbachia* endosymbiont variants.

a, Principal component analysis (PCA) of 57 mitochondrial single-nucleotide polymorphisms (SNPs) from 127 heartworms. Principal component 1 (PC1) and principal component 2 (PC2) explained 20.22% and 11.84% of the variation, respectively. **b,** PCA of 360 *Wolbachia* endosymbiont SNPs from 120 heartworms. PC1 and PC2 explained 33.01% and 15.74% of the variation, respectively. Replicate samples were included in all PCAs. The legend on the right indicates the geographic origin of samples (i.e. COUNTRY: city or region). Abbreviations: USA = United States of America; CRI = Costa Rica; PAN = Panama; ITA = Italy; ROU = Romania; GRC = Greece; THA = Thailand; MYS = Malaysia; AUS = Australia.

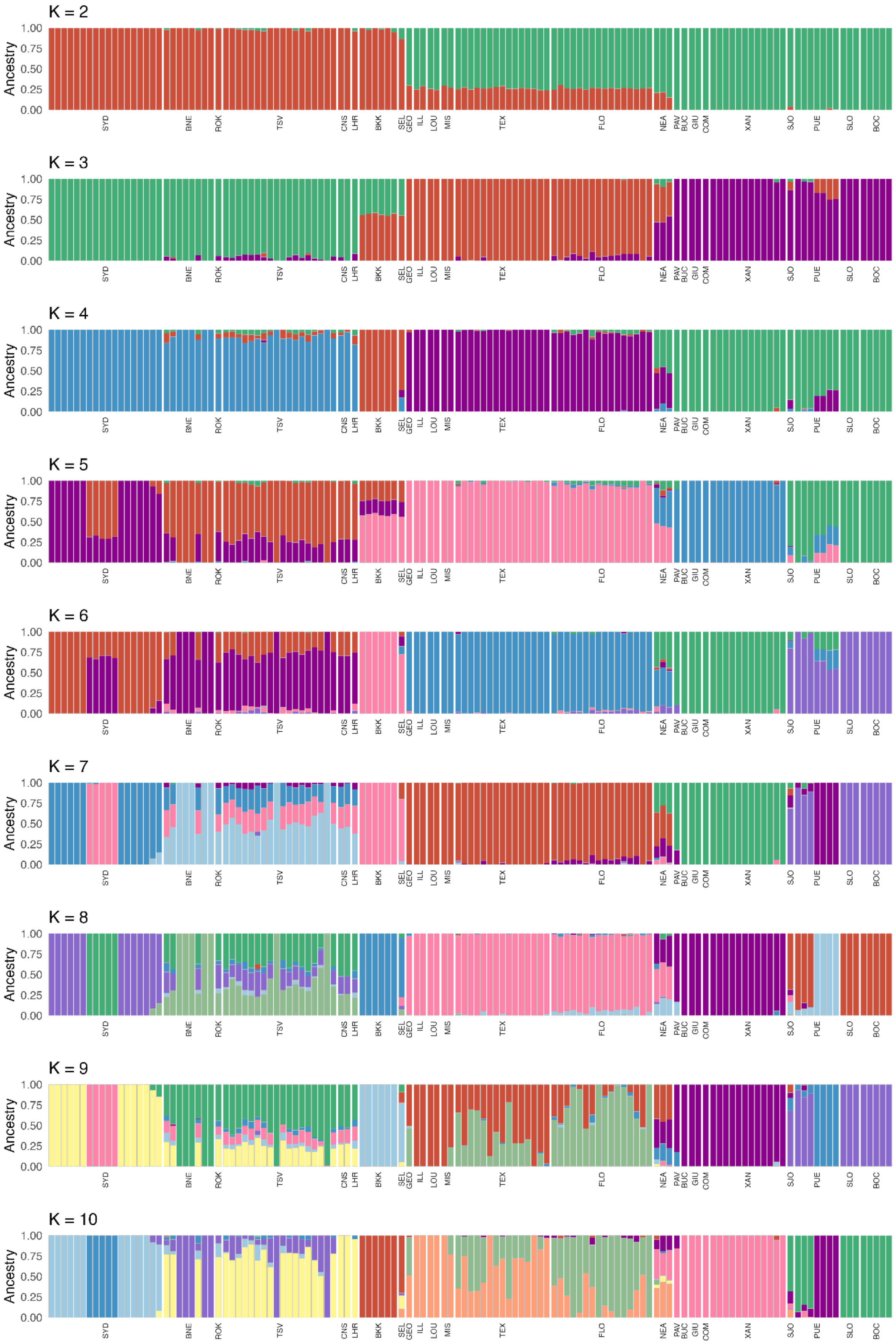

Supplementary Fig. 8: Admixture analysis of adult heartworms.

Analyses were performed in NGSadmix across a range of K values (2 – 10) (colours) for 124 heartworms (vertical bars). Geographic origins are indicated at the bottom of each chart. Abbreviations: Australia (Sydney: SYD, Brisbane:

BNE, Rockhampton: ROK, Townsville: TSV, Cairns: CNS, Lockhart River: LHR); Thailand (Bangkok: BKK); Malaysia (Selangor: SEL); USA (Georgia: GEO, Illinois: ILL, Louisiana: LOU, Missouri: MIS, Texas: TEX, Florida: FLO); Italy (Northeast: NEA, Pavia: PAV); Romania (Bucharest: BUC, Giurgiu: GIU, Comana: COM); Greece (Thessaloniki/Xanthi: XAN); Costa Rica (San José: SJO); Panama (Puerto Armuelles: PUE, San Lorenzo: SLO, Boca Chica: BOC).

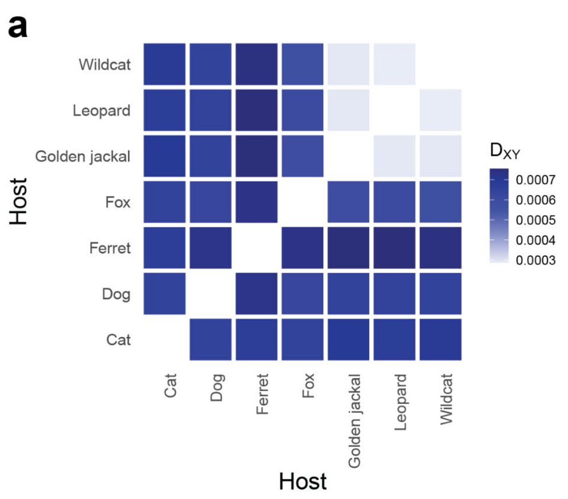

Supplementary Fig. 9: Population genetic summary statistics of heartworms collected from diverse hosts.

The heatmaps show **a,** absolute nucleotide divergence (D_{XY}) and **b,** genetic differentiation (F_{ST}), each calculated using the median of 100 kb sliding windows.

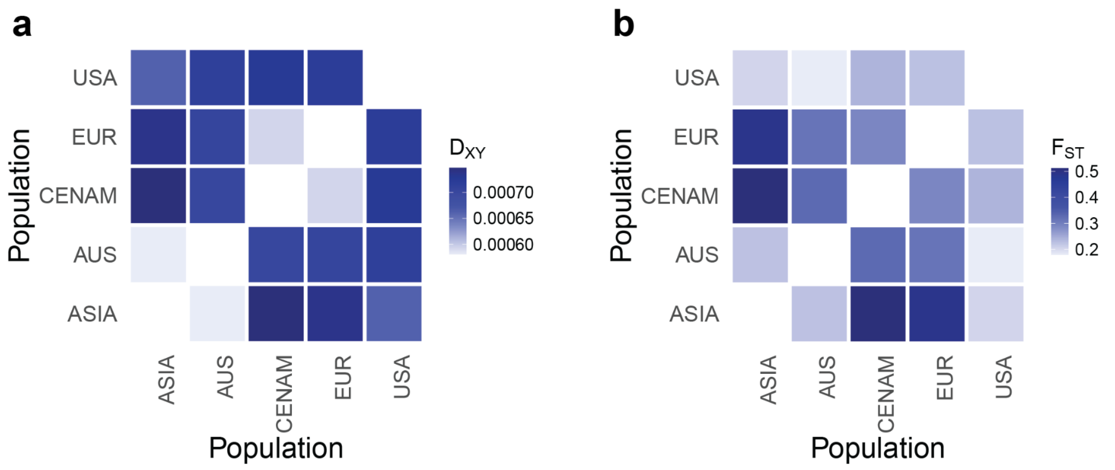

Supplementary Fig. 10: Population genetic summary statistics of heartworms between broad geographical regions.

The heatmaps show **a,** absolute nucleotide divergence (D_{XY}) and **b,** genetic differentiation (F_{ST}), each calculated using the median of 100 kb sliding windows. Region abbreviations: ASIA = Asia; AUS = Australia; CENAM = Central America; EUR = Europe; USA = United States of America.

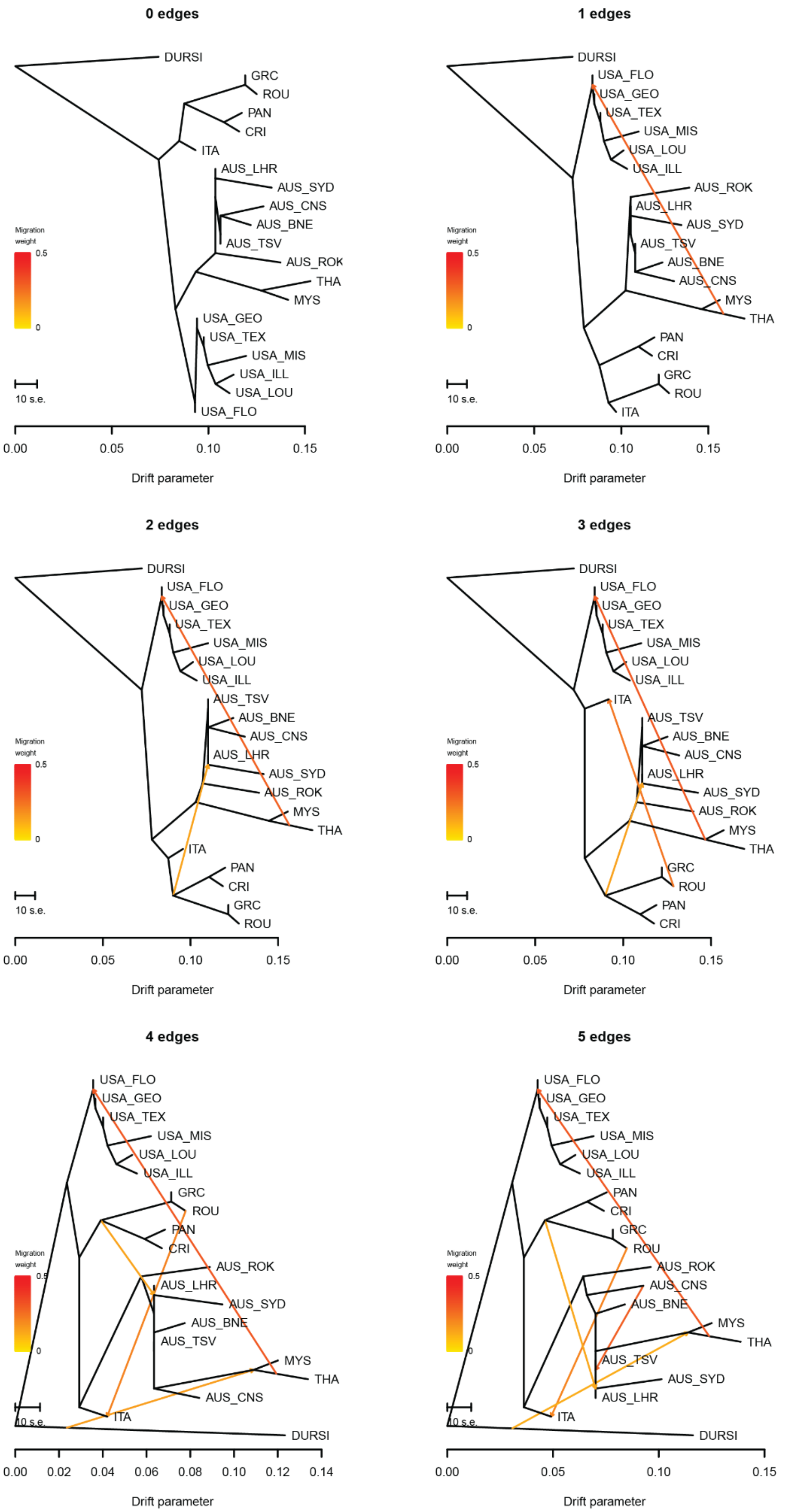
0 edges
DURSI
GRC
ROU
PAN
CRI
ITA
AUS_LHR
AUS_SYD
AUS_CNS
AUS_BNE
AUS_TSV
AUS_ROK
THA
MYS
USA_GEO
USA_TEX
USA_MIS
USA_ILL
USA_LOU
USA_FLO
Migration weight
0.5
0
10 s.e.
0.00 0.05 0.10 0.15
Drift parameter
1 edges
DURSI
USA_FLO
USA_GEO
USA_TEX
USA_MIS
USA_LOU
USA_ILL
AUS_ROK
AUS_LHR
AUS_SYD
AUS_TSV
AUS_BNE
AUS_CNS
MYS
THA
PAN
CRI
GRC
ROU
ITA
Migration weight
0.5
0
10 s.e.
0.00 0.05 0.10 0.15
Drift parameter
2 edges
DURSI
USA_FLO
USA_GEO
USA_TEX
USA_MIS
USA_LOU
USA_ILL
AUS_TSV
AUS_BNE
AUS_CNS
AUS_LHR
AUS_SYD
AUS_ROK
MYS
THA
ITA
PAN
CRI
GRC
ROU
Migration weight
0.5
0
10 s.e.
0.00 0.05 0.10 0.15
Drift parameter
3 edges
DURSI
USA_FLO
USA_GEO
USA_TEX
USA_MIS
USA_LOU
USA_ILL
ITA
AUS_TSV
AUS_BNE
AUS_CNS
AUS_LHR
AUS_SYD
AUS_ROK
MYS
THA
GRC
ROU
PAN
CRI
Migration weight
0.5
0
10 s.e.
0.00 0.05 0.10 0.15
Drift parameter
4 edges
USA_FLO
USA_GEO
USA_TEX
USA_MIS
USA_LOU
USA_ILL
GRC
ROU
PAN
CRI
AUS_ROK
AUS_LHR
AUS_SYD
AUS_BNE
AUS_TSV
MYS
THA
AUS_CNS
ITA
DURSI
Migration weight
0.5
0
10 s.e.
0.00 0.02 0.04 0.06 0.08 0.10 0.12 0.14
Drift parameter
5 edges
USA_FLO
USA_GEO
USA_TEX
USA_MIS
USA_LOU
USA_ILL
PAN
CRI
GRC
ROU
AUS_ROK
AUS_CNS
AUS_BNE
MYS
THA
AUS_TSV
AUS_SYD
AUS_LHR
ITA
DURSI
Migration weight
0.5
0
10 s.e.
0.00 0.05 0.10 0.15
Drift parameter

Supplementary Fig. 11: TreeMix analysis of nuclear heartworm data.

Maximum likelihood trees are shown with migration edges ranging from 0 to 5, using *Dirofilaria ursi* as an outgroup. Coloured lines represent inferred migration events, with the colour indicating the migration weight as an indicator of the level of support for that migration event. Abbreviations: Australia: AUS (Sydney: SYD, Brisbane: BNE, Rockhampton: ROK, Townsville: TSV, Cairns: CNS, Lockhart River: LHR); USA (Georgia: GEO, Illinois: ILL, Louisiana: LOU, Missouri: MIS, Texas: TEX, Florida: FLO); Thailand: THA; Malaysia: MYS; Italy: ITA; Romania: ROU; Greece: GRC; Costa Rica: CRI; Panama: PAN.

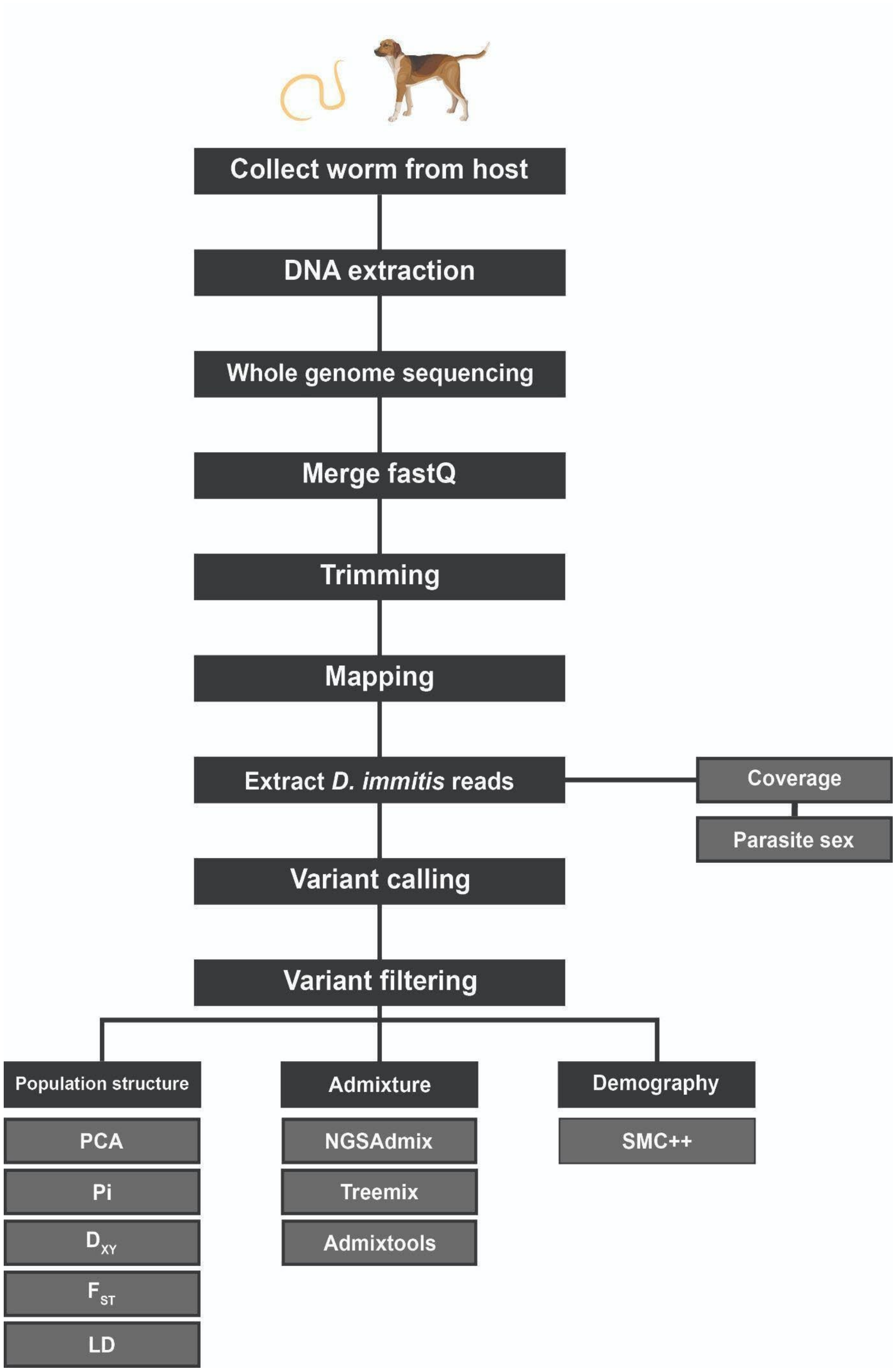

Supplementary Fig. 12: Summary diagram of the population genomics workflow in this study.

Abbreviations of population structure analyses: PCA = Principal Component Analysis; Pi = nucleotide diversity; D_{XY} = absolute nucleotide divergence; F_{ST} = genetic differentiation; LD = linkage disequilibrium. Images used in this figure were obtained from Adobe Stock and are used under license.

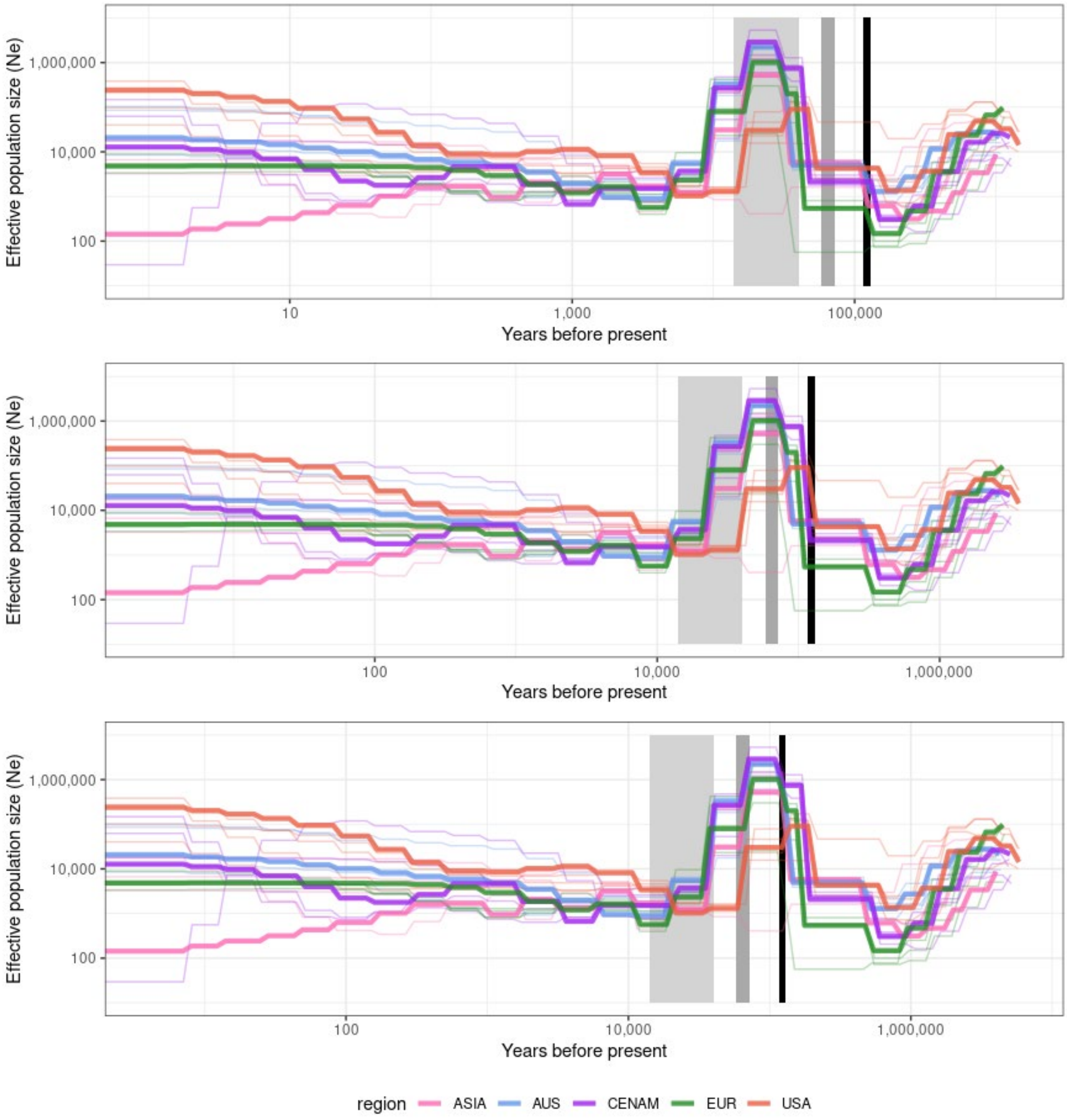

Supplementary Fig. 13: Demographic history analysis of heartworms from dogs using a range of generation times.

Size histories were inferred using SMC++, with each line representing a heartworm subpopulation. A range of generation times was tested for *Dirofilaria immitis* (i.e. 1, 2.5, and 4 years). The lightest grey box indicates previously suggested date ranges for dog domestication (~14–40 kya). The second, darker grey box shows the last interglacial period, and the black box indicates Marine Isotope Stage 4. Abbreviations: AUS = Australia; CENAM = Central America; EUR = Europe; USA = United States of America.
