# Supplementary material for: Population genomics reveals an ancient origin of heartworms in canids
Source: Commun Biol. 2026 Jan 20;9:68. doi: 10.1038/s42003-025-09250-x (PMC12820332; doi:10.1038/s42003-025-09250-x)
Supplement: Supplementary file 2 — Description of Additional Supplementary Materials [file 42003_2025_9250_MOESM2_ESM.pdf]

Description of Additional Supplementary Files

File name: Supplementary Data 1

Description: Genome mapping statistics and coverage for nuclear, mitochondrial, and Wolbachia genomes.

File name: Supplementary Data 2

Description: Literature review of adult heartworm burden in Carnivora hosts

File name: Supplementary Data 3

Description: S ample metadata for global cohort of adult Dirofilaria immitis with outgroups

File name: Supplementary Data 4

Description: Source data underlying the main figures
