# Supplementary material for: Population genomics reveals an ancient origin of heartworms in canids
Source: Commun Biol. 2026 Jan 20;9:68. doi: 10.1038/s42003-025-09250-x (PMC12820332; doi:10.1038/s42003-025-09250-x)
Supplement: Supplementary file 4 — Supplementary Data 2 [file 42003_2025_9250_MOESM4_ESM.docx]

### **Supplementary Data 2: Literature review of adult heartworm burden in Carnivora hosts**

| **Host** | **Location** | **Animals** | **Adult worms /animal** | **Reference** |
| --- | --- | --- | --- | --- |
| **Wolves** | | | | |
| Gray wolves | Italy | 3 | 5 | Moroni et al., 2020 |
| Wolf  (*Canis lupus*) | Serbia | 1 | 37 | Penezić et al., 2014 |
| Wolf  (*Canis lupus*) | Spain | 1 | 1 | Segovia et al., 2001 |
| Grey wolf  (*Canis lupus lupus*) | Serbia | 1 | 42 | Gavrilović et al., 2015 |
| Red wolves  (*Canis rufus gregoryi*) | USA | 8 | 77.8 | Custer & Pence, 1981 |
| **Microfilaremia reported?** | Yes | | | Gomes-de-Sá et al., 2022 |
| **Coyotes** | | | | |
| Coyotes  (*Canis latrans*) | USA | 77 | 21.29 | Aher et al., 2016 |
| Coyotes  (*Canis latrans*) | USA | 36 | 12 | King & Bohning, 1984 |
| Coyotes  (*Canis latrans*) | USA | 2 | 3 | Wixsom et al., 1991 |
| Coyotes  (*Canis latrans*) | USA | 17 | 13.2 | Wixsom et al., 1991 |
| Coyote  (Canis latrans) | USA | 1 | 11 | Kazacos & Edberg, 1979 |
| Coyotes  (*Canis latrans*) | USA | 21 | 19.4 | Sacks, 1998 |
| Coyotes  (*Canis latrans*) | USA | 147 | 8.7 | Nelson et al., 2003 |
| Coyotes  (*Canis latrans*) | USA | 8 | 13.6 | Agostine & Jones, 1982 |
| Coyotes  (*Canis latrans*) | USA | 1 | 5 | Agostine & Jones, 1982 |
| Coyotes  (*Canis latrans*) | USA | 1 | 3 | Agostine & Jones, 1982 |
| Coyotes  (*Canis latrans*) | USA | 20 | 9 | Weinmann & Garcia, 1980 |
| Coyotes  (*Canis latrans*) | USA | 19 | 16.2 | Weinmann & Garcia, 1980 |
| Coyotes  (*Canis latrans*) | USA | 17 | 13.6 | Custer & Pence, 1981 |
| **Microfilaremia reported?** | Yes | | | Weinmann & Garcia, 1980 |
| **Jackals** | | | | |
| Eurasian/golden jackals  (*Canis aureus*) | Iran | 4 | 5.25 | Heidari et al., 2015 |
| Golden jackals  (*Canis aureus*) | Iran | 8 | 6.62 | Sharifdini et al., 2022 |
| Golden jackals  (*Canis aureus*) | Bulgaria | 122 | 4.1 | Panayotova-Pencheva et al., 2016 |
| Jackals  (*Canis aureus*) | Russia | 14 | 12 | Kravchenko et al., 2016 |
| Golden jackals  (*Canis aureus*) | Romania | 12 | 2.92 | Ionică et al., 2022 |
| Golden jackals  (*Canis aureus*) | Romania | 10 | 3 | Ionică et al., 2016 |
| Golden jackals  (*Canis aureus*) | Hungary | 2 | 1 | Tolnai et al., 2014 |
| **Microfilaremia reported?** | Yes | | | Ionică et al., 2016 |
| **Foxes** | | | | |
| Red foxes  (*Vulpes vulpes*) | Spain | 46 | 4.39 | Gortazar et al., 1994 |
| Red foxes  (*Vulpes vulpes*) | Spain | N/A | 4.4 | Gortázar et al., 1998 |
| Gray fox  (*Urocyon cinereoargenteus*) | USA | 2 | 7 | Simmons et al., 1980 |
| Gray fox  (*Urocyon cinereoargenteus*) | USA | 3 | 2 | King & Bohning, 1984 |
| Red fox  (*Vulpes vulpes*) | USA | 1 | 5 | King & Bohning, 1984 |
| Red foxes  (*Vulpes vulpes*) | USA | 5 | 3 | Wixsom et al., 1991 |
| Red foxes  (*Vulpes fulva*) &  Gray foxes  (*Urocyon cinereoargenteus*) | USA | 6 | 5 | Kazacos & Edberg, 1979 |
| Red foxes  (*Vulpes vulpes L.*) | Bulgaria | 29 | 4.79 | Panayotova-Pencheva et al., 2016 |
| Foxes  (*Vulpes vulpes*) | Russia | 48 | 9.2 | Kravchenko et al., 2016 |
| Red foxes  (*Vulpes vulpes*) | Romania | 4 | 1.5 | Ionică et al., 2022 |
| Red Fox  (*Vulpes vulpes*) | Hungary | 20 | 1.5 | Tolnai et al., 2014 |
| **Microfilaremia reported?** | Yes, but the risk that they are a competent reservoir is low. | | | Marks & Bloomfield, 1998  McCall et al., 2008 |
| **Felidae** | | | | |
| Wild cat  (*Felis silvestris*) | Serbia | 1 | 2 | Penezić et al., 2014 |
| Wild cats  (*Felis silvestris*) | Romania | 2 | 1 | Ionică et al., 2022 |
| Cats | USA | 2 | 1.5 | Nelson & Johnson, 2024 |
| Snow leopard (*Uncia uncia*) | Japan | 1 | 3 | Murata et al., 2003 |
| **Microfilaremia reported?** | Infrequently, not considered a competent reservoir. | | | McCall et al., 2008 |
| **Procyonidae** | | | | |
| Raccoon dogs  (*Nyctereutes procyonoides*) | Russia | 28 | 12.6 | Kravchenko et al., 2016 |
| Raccoon dog  (*Nyctereutes procyonoides*) | Romania | 1 | 1 | Ionică et al., 2022 |
| Raccoon dogs  (*Nyctereutes procyonoides viverrinus*) | Japan | 8 | 1.75 | Nakagaki et al., 2000 |
| **Microfilaremia reported?** | Infrequently, not considered a competent reservoir. | | | Ionică et al., 2022 |
| **Mustelidae** | | | | |
| European badger  (*Meles meles*) | Romania | 1 | 3 | Ionică et al., 2022 |
| Ferret (*Mustela putorius furo*) | Hungary | 1 | 2 | Molnár et al., 2010 |
| European badgers (*Meles meles*) | Greece | 2 | 3 | Markakis et al., 2024 |
| **Microfilaremia reported?** | Infrequently, not considered a competent reservoir. | | | McCall et al., 2008 |
| **Pinnipedia** | | | | |
| Seal (*Phoca vitulina*) | Portugal (zoo) | 1 | 32 | Alho et al., 2017 |
| Seal (*Arctocephalus pusillus pusillus*) | Portugal (zoo) | 3 | 17 | Alho et al., 2017 |
| Harbor seal (*Phoca vitulina*) | South Korea (zoo) | 1 | 2 | Kang et al., 2002 |
| **Microfilaremia reported?** | Infrequently, not considered a competent reservoir. | | | Alho et al., 2017 |
| **Ursidae** | | | | |
| Brown bear (*Ursus arctos*) | Greece | 1 | 4 | Papadopoulos et al., 2017 |
| Black bear (*Ursus americanus*) | USA | 4 | 3 | Crum et al., 1978 |
| Black bear (*Ursus americanus*) | USA | 1 | 5 | Johnson, 1975 |
| **Microfilaremia reported?** | Infrequently, not considered a competent reservoir. | | | McCall et al., 2008 |
| **Dogs** | | | | |
| Dogs | Iran | 10 | 23.5 | Sharifdini et al., 2022 |
| Dogs | Bulgaria | 9 | 14.43 | Panayotova-Pencheva et al., 2016 |
| Dog | USA | 1 | 150 | Oliveira et al., 2021 |
| Dogs | Italy | 2 | 15.5 | Santoro et al., 2019 |
| Dogs | Taiwan | 477 | 7.2 | Wu & Fan, 2003 |
| Dogs | Mexico | 52 | 4.5 | Bolio-Gonzalez et al., 2007 |
| Dogs | USA | 170 | 14 | Kaiser & Williams, 2004 |
| Dogs | Australia | 15 | 5.8 | Bidgood & Collins, 1996 |
| Dogs | Hungary | 2 | 9 | Tolnai et al., 2014 |
| Dogs | N/A | 6 | 23.83 | Rafailov et al., 2022 |
| Dogs | USA | 50 | 7.62 | Henry et al., 2018 |
| Dogs | USA | 50 | 24.62 | Henry et al., 2018 |
| Dogs | USA | 50 | 53.86 | Henry et al., 2018 |
| Dogs | USA | 50 | 104.92 | Henry et al., 2018 |
| Dogs | Panama | 3 | 44 | Chacón & Candanedo, 2021 |
| **Microfilaremia reported?** | Yes | | | Panetta et al., 2021 |

**References**

Agostine, J. C., & Jones, G. S. (1982). Heartworms (*Dirofilaria immitis*) in coyotes (*Canis latrans*) in New England. *J Wildl Dis*, *18*(3), 343-345. https://doi.org/10.7589/0090-3558-18.3.343

Aher, A. M., Caudill, D., Caudill, G., Butryn, R. S., Wolf, D., Fox, M., Blake, D. P., & Cunningham, M. W. (2016). Prevalence, genetic analyses, and risk factors associated with heartworm (*Dirofilaria immitis*) in wild coyotes (*Canis latrans*) from Florida, USA. *J Wildl Dis*, *52*(4), 785-792. https://doi.org/10.7589/2015-09-223

Alho, A. M., Marcelino, I., Colella, V., Flanagan, C., Silva, N., Correia, J. J., Latrofa, M. S., Otranto, D., & Madeira de Carvalho, L. (2017). *Dirofilaria immitis* in pinnipeds and a new host record. *Parasit Vectors*, *10*(1), 142. https://doi.org/10.1186/s13071-017-2073-0

Bidgood, A., & Collins, G. H. (1996). The prevalence of *Dirofilaria immitis* in dogs in Sydney. *Aus Vet J*, *73*(3), 103-104. https://doi.org/10.1111/j.1751-0813.1996.tb09987.x

Bolio-Gonzalez, M. E., Rodriguez-Vivas, R. I., Sauri-Arceo, C. H., Gutierrez-Blanco, E., Ortega-Pacheco, A., & Colin-Flores, R. F. (2007). Prevalence of the *Dirofilaria immitis* infection in dogs from Merida, Yucatan, Mexico. *Vet Parasitol*, *148*(2), 166-169. https://doi.org/10.1016/j.vetpar.2007.05.019

Chacón, S., & Candanedo, P. (2021). Canine dirofilariasis in Panama first cases report immunochromatography and necropsy diagnosis. *J Appl Microbiol Res*, *4*(1), 7-17.

Crum, J. M., Nettles, V. F., & Davidson, W. R. (1978). Studies on endoparasites of the black bear (*Ursus americanus*) in the southeastern United States. *J Wildl Dis*, *14*(2), 178-186. https://doi.org/10.7589/0090-3558-14.2.178

Custer, J. W., & Pence, D. B. (1981). Dirofilariasis in wild canids from the Gulf coastal prairies of Texas and Louisiana, U.S.A. *Vet Parasitol*, *8*(1), 71-82. https://doi.org/10.1016/0304-4017(81)90019-4

Gavrilović, P., Blitva-Robertson, G., Özvegy, J., Kiskároly, F., & Becskei, Z. (2015). Case report of dirofilariasis in grey wolf in Serbia. *Acta Parasitol*, *60*(1), 175-178. https://doi.org/10.1007/BF03406010

Gomes-de-Sá, S., Santos-Silva, S., Moreira, A. S., Barradas, P. F., Amorim, I., Cardoso, L., & Mesquita, J. R. (2022). *Dirofilaria immitis* antigenemia and microfilaremia in Iberian wolves and red foxes from Portugal. *Parasit Vectors*, *15*, 119. https://doi.org/10.1186/s13071-022-05170-5

Gortazar, C., Castillo, J. A., Lucientes, J., Blanco, J. C., Arriolabengoa, A., & Calvete, C. (1994). Factors affecting *Dirofilaria immitis* prevalence in red foxes in northeastern Spain. *J Wildl Dis*, *30*(4), 545-547. https://doi.org/10.7589/0090-3558-30.4.545

Gortázar, C., Villafuerte, R., Lucientes, J., & Fernández-de-Luco, D. (1998). Habitat related differences in helminth parasites of red foxes in the Ebro valley. *Vet Parasitol*, *80*(1), 75-81. https://doi.org/10.1016/S0304-4017(98)00192-7

Heidari, Z., Kia, E. B., Arzamani, K., Sharifdini, M., Mobedi, I., Zarei, Z., & Kamranrashani, B. (2015). Morphological and molecular identification of *Dirofilaria immitis* from Jackal (*Canis aureus*) in North Khorasan, northeast Iran. *J Vector Borne Dis*, *52*(4), 329-333.

Henry, L. G., Brunson, K. J., Walden, H. S., Wenzlow, N., Beachboard, S. E., L Barr, K., & Long, M. T. (2018). Comparison of six commercial antigen kits for detection of *Dirofilaria immitis* infections in canines with necropsy-confirmed heartworm status. *Vet Parasitol*, *254*, 178-182. https://doi.org/10.1016/j.vetpar.2018.02.037

Ionică, A. M., Deak, G., Boncea, R., Gherman, C. M., & Mihalca, A. D. (2022). The European Badger as a new host for *Dirofilaria immitis* and an update on the distribution of the heartworm in wild carnivores from Romania. *Pathogens*, *11*(4), 420. https://doi.org/10.3390/pathogens11040420

Ionică, A. M., Matei, I. A., D'Amico, G., Daskalaki, A. A., Juránková, J., Ionescu, D. T., Mihalca, A. D., Modrý, D., & Gherman, C. M. (2016). Role of golden jackals (*Canis aureus*) as natural reservoirs of *Dirofilaria* spp. in Romania. *Parasit Vectors*, *9*, 240. https://doi.org/10.1186/s13071-016-1524-3

Johnson, C. A. (1975). *Ursus americanus* (Black Bear) a new host for *Dirofilaria immitis*. *J Parasitol*, *61*(5), 940. https://doi.org/10.2307/3279242

Kaiser, L., & Williams, J. F. (2004). *Dirofilaria immitis*: worm burden and pulmonary artery proliferation in dogs from Michigan (United States). *Vet Parasitol*, *124*(1), 125-129. https://doi.org/10.1016/j.vetpar.2004.06.015

Kang, S.-s., Kim, J.-h., Kwon, Y.-b., & Park, S.-h. (2002). A harbor seal infection with *Dirofilaria*. *J Vet Clin*, *19*(1), 92-94.

Kazacos, K. R., & Edberg, E. O. (1979). *Dirofilaria immitis* infection in foxes and coyotes in Indiana. *JAVMA*, *175*(9), 909-910.

King, A. W., & Bohning, A. M. (1984). The incidence of heartworm *Dirofilaria immitis* (Filarioidea), in the wild canids of northeast Arkansas. *Southw Nat*, *29*(1), 89-92. https://doi.org/10.2307/3670773

Kravchenko, V., Itin, G., Kartashev, V., Ermakov, A., Kartashov, S., Diosdado, A., González-Miguel, J., & Simón, F. (2016). *Dirofilaria immitis* and *D. repens* in sylvatic reservoirs of Krasnodar Krai (Russian Federation). *Vet Parasitol Reg Stud Reports*, *6*, 35-38. https://doi.org/10.1016/j.vprsr.2016.08.004

Markakis, G., Sioutas, G., Bitchava, D., Komnenou, A., Ganoti, M., & Papadopoulos, E. (2024). Is the European badger a new host for *Dirofilaria immitis*? The first records in Greece. *Parasitol Res*, *123*(2), 118. https://doi.org/10.1007/s00436-024-08141-0

Marks, C. A., & Bloomfield, T. E. (1998). Canine heartworm (*Dirofilaria immitis*) detected in red foxes (*Vulpes vulpes*) in urban Melbourne. *Vet Parasitol*, *78*(2), 147-154. https://doi.org/10.1016/S0304-4017(98)00131-9

McCall, J. W., Genchi, C., Kramer, L. H., Guerrero, J., & Venco, L. (2008). Heartworm disease in animals and humans. *Adv Parasitol, 66*, 193-285. https://doi.org/10.1016/S0065-308X(08)00204-2

Molnár, V., Pazár, P., Rigó, D., Máthé, D., Fok, É., Glávits, R., Vajdovich, P., Jacsó, O., Balogh, L., & Sós, E. (2010). Autochthonous *Dirofilaria immitis* infection in a ferret with aberrant larval migration in Europe. *J Small Anim Pract*, *51*(7), 393-396. https://doi.org/10.1111/j.1748-5827.2010.00950.x

Moroni, B., Rossi, L., Meneguz, P. G., Orusa, R., Zoppi, S., Robetto, S., Marucco, F., & Tizzani, P. (2020). *Dirofilaria immitis* in wolves recolonizing northern Italy: are wolves competent hosts? *Parasit Vectors*, *13*, 482. https://doi.org/10.1186/s13071-020-04353-2

Murata, K., Yanai, T., Agatsuma, T., & Uni, S. (2003). *Dirofilaria immitis* infection of a Snow Leopard (*Uncia uncia*) in a Japanese Zoo with mitochondrial DNA analysis. *J Vet Med Sci*, *65*(8), 945-947. https://doi.org/10.1292/jvms.65.945

Nakagaki, K., Suzuki, T., Hayama, S. I., & Kanda, E. (2000). Prevalence of dirofilarial infection in raccoon dogs in Japan. *Parasitol Int*, *49*(3), 253-256. https://doi.org/10.1016/S1383-5769(00)00049-0

Nelson, C. T., & Johnson, C. M. (2024). Evaluation of feline heartworm disease based on gross necropsy, serology, pulmonary histopathology, and radiographic evidence in adult shelter cats in northeastern Alabama. *Parasit Vectors*, *17*(1), 161. https://doi.org/10.1186/s13071-024-06178-9

Nelson, T. A., Gregory, D. G., & Laursen, J. R. (2003). Canine heartworms in coyotes in Illinois. *J Wildl Dis*, *39*(3), 593-599. https://doi.org/10.7589/0090-3558-39.3.593

Oliveira, L. B., McHale, B. J., Verocai, G. G., & Rissi, D. R. (2021). Subcutaneous and cardiopulmonary dirofilariasis in a dog. *Can Vet J*, *62*(8), 854-856.

Panayotova-Pencheva, M. S., Mirchev, R. L., & Trifonova, A. P. (2016). *Dirofilaria immitis* infection in carnivores from Bulgaria: 2012–2013 update. *Bulg J Vet Med*, *19*(2), 153-162. https://doi.org/10.15547/bjvm.918

Panetta, J. L., Calvani, N. E. D., Orr, B., Nicoletti, A. G., Ward, M. P., & Šlapeta, J. (2021). Multiple diagnostic tests demonstrate an increased risk of canine heartworm disease in northern Queensland, Australia. *Parasit Vectors*, *14*, 393. https://doi.org/10.1186/s13071-021-04896-y

Papadopoulos, E., Komnenou, A., Poutachides, T., Heikkinen, P., Oksanen, A., & Karamanlidis, A. A. (2017). Detection of *Dirofilaria immitis* in a brown bear (*Ursus arctos*) in Greece. *Helminthologia*, *54*(3), 257-261. https://doi.org/10.1515/helm-2017-0033

Penezić, A., Selaković, S., Pavlović, I., & Ćirović, D. (2014). First findings and prevalence of adult heartworms (*Dirofilaria immitis*) in wild carnivores from Serbia. *Parasitol Res*, *113*, 3281-3285. https://doi.org/10.1007/s00436-014-3991-9

Rafailov, R., Popov, G., Kanchev, K., & Manov, V. (2022). Pathomorphological findings in dogs with spontaneous hearthworm disease. *Tradit Mod Vet Med*, *7*(1), 53-59.

Sacks, B. N. (1998). Increasing prevalence of canine heartworm in coyotes from California. *J Wildl Dis*, *34*(2), 386-389. https://doi.org/10.7589/0090-3558-34.2.386

Santoro, M., Miletti, G., Vangone, L., Spadari, L., Reccia, S., & Fusco, G. (2019). Heartworm disease (*Dirofilaria immitis*) in two roaming dogs from the urban area of Castel Volturno, Southern Italy. *Front Vet Sci*, *6*, 270. https://doi.org/10.3389/fvets.2019.00270

Segovia, J. M., Torres, J., Miquel, J., Llaneza, L., & Feliu, C. (2001). Helminths in the wolf, *Canis lupus*, from north-western Spain. *J Helminthol*, *75*(2), 183-192. https://doi.org/10.1079/JOH200152

Sharifdini, M., Karimi, M., Ashrafi, K., Soleimani, M., & Mirjalali, H. (2022). Prevalence and molecular characterization of *Dirofilaria immitis* in road killed canids of northern Iran. *BMC Vet Res*, *18*(1), 161. https://doi.org/10.1186/s12917-022-03270-z

Simmons, J. M., Nicholson, W. S., Hill, E. P., & Briggs, D. B. (1980). Occurance of (*Dirofilaria immitis*) in gray fox (*Urocyon cinereoargenteus*) in Alabama and Georgia. *J Wildl Dis*, *16*(2), 225-228. https://doi.org/10.7589/0090-3558-16.2.225

Tolnai, Z., Széll, Z., Sproch, Á., Szeredi, L., & Sréter, T. (2014). *Dirofilaria immitis*: An emerging parasite in dogs, red foxes and golden jackals in Hungary. *Vet Parasitol*, *203*(3), 339-342. https://doi.org/10.1016/j.vetpar.2014.04.004

Weinmann, C. J., & Garcia, R. (1980). Coyotes and canine heartworm in California. *J Wildl Dis*, *16*(2), 217-221. https://doi.org/10.7589/0090-3558-16.2.217

Wixsom, M. J., Green, S. P., Corwin, R. M., & Fritzell, E. K. (1991). *Dirofilaria immitis* in coyotes and foxes in Missouri. *J Wildl Dis*, *27*(1), 166-169. https://doi.org/10.7589/0090-3558-27.1.166

Wu, C. C., & Fan, P. C. (2003). Prevalence of canine dirofilariasis in Taiwan. *J Helminthol*, *77*(1), 83-88. https://doi.org/10.1079/joh2002150
