# Supplementary material for: Population genomics reveals an ancient origin of heartworms in canids
Source: Commun Biol. 2026 Jan 20;9:68. doi: 10.1038/s42003-025-09250-x (PMC12820332; doi:10.1038/s42003-025-09250-x)
Supplement: Supplementary file 7 — Reporting Summary [file 42003_2025_9250_MOESM7_ESM.pdf]

nature portfolio

Corresponding author(s): Jan Slapeta and Stephen R. Doyle

Last updated by author(s): Nov 3, 2025

Reporting Summary

Nature Portfolio wishes to improve the reproducibility of the work that we publish. This form provides structure for consistency and transparency in reporting. For further information on Nature Portfolio policies, see our Editorial Policies and the Editorial Policy Checklist.

Statistics

For all statistical analyses, confirm that the following items are present in the figure legend, table legend, main text, or Methods section.

n/a	Confirmed	
☐	☒	The exact sample size (*n*) for each experimental group/condition, given as a discrete number and unit of measurement
☐	☒	A statement on whether measurements were taken from distinct samples or whether the same sample was measured repeatedly
☐	☒	The statistical test(s) used AND whether they are one- or two-sided<br>*Only common tests should be described solely by name; describe more complex techniques in the Methods section.*
☒	☐	A description of all covariates tested
☒	☐	A description of any assumptions or corrections, such as tests of normality and adjustment for multiple comparisons
☐	☒	A full description of the statistical parameters including central tendency (e.g. means) or other basic estimates (e.g. regression coefficient) AND variation (e.g. standard deviation) or associated estimates of uncertainty (e.g. confidence intervals)
☐	☒	For null hypothesis testing, the test statistic (e.g. F, t, r) with confidence intervals, effect sizes, degrees of freedom and P value noted<br>*Give P values as exact values whenever suitable.*
☒	☐	For Bayesian analysis, information on the choice of priors and Markov chain Monte Carlo settings
☒	☐	For hierarchical and complex designs, identification of the appropriate level for tests and full reporting of outcomes
☒	☐	Estimates of effect sizes (e.g. Cohen's *d*, Pearson's *r*), indicating how they were calculated

Our web collection on statistics for biologists contains articles on many of the points above.

Software and code

Policy information about availability of computer code

Data collection	No software was used to collect data in this study.
Data analysis	The code used to analyse the data in this study is available from GitHub: https://github.com/rosemondepower/heartworm_genome.git. Bioinformatics tools used for analysis include Sratoolkit version 3.0.10, Wget version 1.21.2, FastQC version 0.11.8, MultiQC version 1.17, Trim Galore version 0.4.4, Nextflow mapping pipeline (mapping-helminth/v1.0.9) using Minimap2 (version 2.17) as a mapper and GATK (version 4.1.4.1), Samtools (version 1.14) and Sambamba (version 1.0) for further data processing, BamTools (version 2.5.1), BEDTools makewindows (version 2.31.0), SAMtools bedcov, GATK (version 4.1.4.1), VCFtools (version 0.1.16), SNPRelate (version 1.38.0), pixy (version 2.0.0.beta12), NGSadmix from the ANGSD package, CLUMPAK, TreeMix (version 1.13), Custom scripts: 'ldPruning.sh' and 'vcf2treemix.sh' obtained from https://github.com/speciationgenomics/scripts; 'convertVCFtoEigenstrat.sh' obtained from https://github.com/speciationgenomics/scripts/, OptM (version 0.1.8; https://github.com/cran/OptM), AdmixTools (version 8.0.2), PLINK (version 1.90b6.18), and SMC++ (version 1.15.4).

For manuscripts utilizing custom algorithms or software that are central to the research but not yet described in published literature, software must be made available to editors and reviewers. We strongly encourage code deposition in a community repository (e.g. GitHub). See the Nature Portfolio guidelines for submitting code & software for further information.

Data

Policy information about availability of data

All manuscripts must include a data availability statement. This statement should provide the following information, where applicable:

- Accession codes, unique identifiers, or web links for publicly available datasets
- A description of any restrictions on data availability
- For clinical datasets or third party data, please ensure that the statement adheres to our policy

Raw FastQ sequences generated in this study are available at SRA NCBI BioProject PRJNA1104412.

Research involving human participants, their data, or biological material

Policy information about studies with human participants or human data. See also policy information about sex, gender (identity/presentation), and sexual orientation and race, ethnicity and racism.

Reporting on sex and gender	not applicable
Reporting on race, ethnicity, or other socially relevant groupings	not applicable
Population characteristics	not applicable
Recruitment	not applicable
Ethics oversight	not applicable

Note that full information on the approval of the study protocol must also be provided in the manuscript.

Field-specific reporting

Please select the one below that is the best fit for your research. If you are not sure, read the appropriate sections before making your selection.

☐ Life sciences ☐ Behavioural & social sciences ☒ Ecological, evolutionary & environmental sciences

For a reference copy of the document with all sections, see nature.com/documents/nr-reporting-summary-flat.pdf

Ecological, evolutionary & environmental sciences study design

All studies must disclose on these points even when the disclosure is negative.

Study description	Using whole genome sequencing of opportunistically collected adult worms obtained from nine countries on four continents, we analyse the diversity, genetic structure, and population demographics of modern heartworms to infer their historical origins and dispersal.
Research sample	A total of 120 individual adult Dirofilaria immitis were obtained from Australia (n = 43), the USA (n = 37), Panama (n = 14), Greece (n = 12), Thailand (n = 6), Italy (n = 3), Romania (n = 3), Costa Rica (n = 1), and Malaysia (n = 1). The sampling regime aimed to collect adult D. immitis from a wide geographical range. Most of these samples were obtained from dogs (Canis lupus familiaris; n = 115) but also included some from cats (Felis catus; n = 4), foxes (Vulpes vulpes; n = 4), a ferret (Mustela putorius furo; n = 1), a golden jackal (Canis aureus; n = 1), a leopard (Panthera pardus; n = 1), and a wildcat (Felis silvestris; n = 1) (metadata for all samples is presented in Supplementary Table 3). We included five technical replicates (Brisbane: n = 1; Sydney: n = 2; Malaysia: n = 1; Panama: n = 1). To provide outgroup samples for our analyses, we collected adult D. repens (n = 2) from dogs in Italy, Dirofilaria sp. 'Thailand II' (n = 2) from humans (Homo sapiens) in Vietnam and D. ursi (n = 1) from an American black bear (Ursus americanus) in the USA.
Sampling strategy	Opportunistic during postmortems.
Data collection	Heartworms were collected by coauthors and shipped to the University of Sydney where they were assigned an accession number and meta-data stored according to the University Research Data Management plan.
Timing and spatial scale	2020-2024
Data exclusions	No primary data excluded
Reproducibility	We have repeated 5 DNA isolations from the same specimen to verify our results.

Randomization	Randomization of samples was not relevant as we aimed for the maximum number of heartworms from as many countries as possible
Blinding	We aimed at maximum number of heartworms from as many countries as possible

Did the study involve field work? ☐ Yes ☒ No

Reporting for specific materials, systems and methods

We require information from authors about some types of materials, experimental systems and methods used in many studies. Here, indicate whether each material, system or method listed is relevant to your study. If you are not sure if a list item applies to your research, read the appropriate section before selecting a response.

Materials & experimental systems

n/a	Involved in the study
☒	☐ Antibodies
☒	☐ Eukaryotic cell lines
☒	☐ Palaeontology and archaeology
☐	☒ Animals and other organisms
☒	☐ Clinical data
☒	☐ Dual use research of concern
☒	☐ Plants

Methods

n/a	Involved in the study
☒	☐ ChIP-seq
☒	☐ Flow cytometry
☒	☐ MRI-based neuroimaging

Animals and other research organisms

Policy information about studies involving animals; ARRIVE guidelines recommended for reporting animal research, and Sex and Gender in Research

Laboratory animals	Not applicable
Wild animals	Material collected opportunistically from deceased animals at post mortem
Reporting on sex	Parasites sex was recorded and verified using genomics
Field-collected samples	Material collected opportunistically from deceased animals at post mortem
Ethics oversight	Material collected from dead animals at post mortem, collection of parasites at post mortem does not require ethics approval

Note that full information on the approval of the study protocol must also be provided in the manuscript.

Plants

Seed stocks	Not applicable
Novel plant genotypes	Not applicable
Authentication	Not applicable
